# Implementation of Electronic Informed Consent in Biomedical Research and Stakeholders’ Perspectives: Systematic Review

**DOI:** 10.2196/19129

**Published:** 2020-10-08

**Authors:** Evelien De Sutter, Drieda Zaçe, Stefania Boccia, Maria Luisa Di Pietro, David Geerts, Pascal Borry, Isabelle Huys

**Affiliations:** 1 Clinical Pharmacology and Pharmacotherapy Department of Pharmaceutical and Pharmacological Sciences KU Leuven Leuven Belgium; 2 Section of Hygiene University Department of Life Sciences and Public Health Università Cattolica del Sacro Cuore Roma Italy; 3 Department of Woman and Child Health and Public Health Fondazione Policlinico Universitario A Gemelli IRCCS Roma Italy; 4 Meaningful Interactions Lab KU Leuven Leuven Belgium; 5 Centre for Biomedical Ethics and Law Department of Public Health and Primary Care KU Leuven Leuven Belgium

**Keywords:** Informed consent, systematic review, biomedical research, user interface, research ethics, digital health

## Abstract

**Background:**

Informed consent is one of the key elements in biomedical research. The introduction of electronic informed consent can be a way to overcome many challenges related to paper-based informed consent; however, its novel opportunities remain largely unfulfilled due to several barriers.

**Objective:**

We aimed to provide an overview of the ethical, legal, regulatory, and user interface perspectives of multiple stakeholder groups in order to assist responsible implementation of electronic informed consent in biomedical research.

**Methods:**

We conducted a systematic literature search using Web of Science (Core collection), PubMed, EMBASE, ACM Digital Library, and PsycARTICLES. PRISMA (Preferred Reporting Items for Systematic Reviews and Meta-Analyses) guidelines were used for reporting this work. We included empirical full-text studies focusing on the concept of electronic informed consent in biomedical research covering the ethical, legal, regulatory, and user interface domains. Studies written in English and published from January 2010 onward were selected. We explored perspectives of different stakeholder groups, in particular researchers, research participants, health authorities, and ethics committees. We critically appraised literature included in the systematic review using the Newcastle-Ottawa scale for cohort and cross-sectional studies, Critical Appraisal Skills Programme for qualitative studies, Mixed Methods Appraisal Tool for mixed methods studies, and Jadad tool for randomized controlled trials.

**Results:**

A total of 40 studies met our inclusion criteria. Overall, the studies were heterogeneous in the type of study design, population, intervention, research context, and the tools used. Most of the studies’ populations were research participants (ie, patients and healthy volunteers). The majority of studies addressed barriers to achieving adequate understanding when using electronic informed consent. Concerns shared by multiple stakeholder groups were related to the security and legal validity of an electronic informed consent platform and usability for specific groups of research participants.

**Conclusions:**

Electronic informed consent has the potential to improve the informed consent process in biomedical research compared to the current paper-based consent. The ethical, legal, regulatory, and user interface perspectives outlined in this review might serve to enhance the future implementation of electronic informed consent.

**Trial Registration:**

PROSPERO International Prospective Register of Systematic Reviews CRD42020158979; https://www.crd.york.ac.uk/prospero/display_record.php?RecordID=158979

## Introduction

Obtaining informed consent is a fundamental ethical practice in biomedical research. It is the process of providing meaningful information to the potential participant in order to enable an autonomous well-informed decision on whether or not they wish to participate in the research study [1-3]. Moreover, informed consent may serve as one of the legal grounds for processing personal data, as described in articles 6 and 9 of the General Data Protection Regulation [4]. The primary goal of informed consent is to truly inform potential research participants or their representatives about different aspects such as the study design, study procedures, the risks and benefits, treatment options, participants’ responsibilities, and the right to withdraw as well as information regarding data processing [1,3,4]. Therefore, information must be available in lay terminology and in a language understandable to the participants [3]. Long and cumbersome paper-based informed consent documents are the result of the increasing complexity of clinical research and the multitude of legal and regulatory requirements to satisfy informed consent needs [5-7]. Regulatory requirements refer to those related to the regulatory approval of medicines [8]. Available evidence has shown that research participants lack understanding of the key concepts of research studies [7,9]. For this reason, many attempts have been made to improve the understanding of research participants [10].

Owing to innovations in information technology, different strategies to consent have been developed, ranging from involving multimedia to the implementation of quizzes [10]. Research on the use of different multimedia formats to present information and improve research participants’ understanding is gaining popularity [10-12]. Recently, the US Food and Drug Administration (FDA), in collaboration with the Office for Human Research Protections, issued guidance [13] concerning electronic informed consent in order to provide a shared and harmonized approach. Electronic informed consent refers to electronic systems which may incorporate multimedia in order to convey information and to obtain informed consent. In this guidance [13], recommendations are described covering several aspects related to electronic informed consent such as the presentation of information, the use of electronic signatures, identity verification, FDA inspections, and the review process by ethics committees. The development of an electronic informed consent platform, enabling participants to give and manage their electronic informed consent, could offer several opportunities. First, it could facilitate long-term interaction with research participants in cases where reconsenting for follow-up studies is required or for providing research results. Second, it may truly inform research participants in an interactive, tailored approach based on the individual’s information needs [14].

Considerable research has been devoted to single aspects important for electronic informed consent; however, rather less attention has been paid to integrating information from several important scientific domains such as ethical, legal, regulatory, and user interface domains. It is vital to balance the relevant domains in order to create an electronic informed consent platform that better informs, empowers, and engages research participants [15,16]. In the field of research, the ethics committee plays an important role as it is responsible for reviewing study protocols to ensure that they meet the ethical, legal, and regulatory requirements of the country where the research is being conducted, paying attention to the applicability of international norms and standards [3]. However, it remains unclear to what extent ethics committees are familiar with electronic informed consent and how they will handle electronic informed consent. Moreover, the involvement of research participants in the design of an electronic informed consent platform is of utmost importance as they fulfill a central role in biomedical research. Personalized human-centered design enables understanding of the participants’ experiences and incorporation of their feedback to facilitate a participant-centered electronic informed consent platform [17].

Despite an increasing number of studies relevant for electronic informed consent, a comprehensive overview of these studies across the ethical, legal, regulatory, and user interface domains is lacking. Hence, the primary outcome of this systematic review was to provide a descriptive overview of the perspectives of different stakeholder groups (ie, researchers, research participants, health authorities, and ethics committee members) in these different domains with regard to electronic informed consent in biomedical research. The secondary outcome aimed to provide recommendations to assist responsible implementation. Insights of this review may serve as the foundation to design an electronic informed consent platform, thereby taking scientific steps forward in view of the international state-of-the-art.

## Methods

This systematic review was reported according to the Preferred Reporting Items for Systematic Reviews and Meta-Analyses (PRISMA) statement [18]. The corresponding review protocol is registered in the International Prospective Register of Systematic Reviews (PROSPERO CRD42020158979).

### Search Strategy

The electronic databases of Web of Science (Core collection), PubMed, EMBASE, ACM Digital Library, and PsycARTICLES were searched in order to retrieve potential eligible studies. The searches for all databases were performed November 14, 2019. A search string for PubMed was developed and consisted of Medical Subject Headings terms and free-text words. The search string was adjusted for use in the other electronic databases. All search strings were verified by a health sciences librarian. These search strings consisted of keywords such as *electronic (informed) consent*, *dynamic (informed) consent*, *e-consent*, *digital (informed) consent*, *interactive (informed) consent*, *online (informed) consent*, *multimedia*, and *telemedicine*. The search was restricted to studies published in English after January 1, 2010. This timeframe was justified by the fact that electronic informed consent in biomedical research gained popularity only in the last decade and that technology has evolved quite rapidly. The full search strategy for all databases can be found in Multimedia Appendix 1.

### Study Selection and Criteria

All study types (ie, qualitative, quantitative, and mixed methods) that discussed the concept of electronic informed consent in biomedical research covering the ethical, legal, regulatory, and user interface domains were included. Perspectives of stakeholders, in particular research participants (ie, patients and healthy volunteers), researchers, ethics committee members, and health authorities were considered relevant. We excluded nonempirical studies and abstracts. All studies retrieved from the search strategy were imported to EndNote X9 (Clarivate Analytics) and duplicates were removed. The remaining studies were uploaded to Rayyan (Qatar Computing Research Institute) software. Two researchers (EDS and DZ) independently performed the first screening based on titles and abstracts. In a second step, studies with full texts available were carefully reviewed by two researchers (EDS and DZ) and disagreements were resolved by consensus. When the full texts were not available, the corresponding authors were contacted. The reference lists of the included studies were hand searched for additional studies.

### Data Extraction and Analysis

Data extraction was performed independently by two researchers (EDS and DZ) and was subsequently checked. A dedicated Excel (Microsoft Inc) data extraction form was used retrieving the following information for each eligible study: study identification (first author, title, publication year); study characteristics (study period, country, design, objective, scenario); stakeholder group (research participants, researchers, ethics committee members, health authorities); tool used to collect information (survey, focus groups, interviews); intervention (a description of the electronic informed consent platform); domain being assessed (ethical, legal, regulatory, or user interface); and ethical, legal, regulatory or user interface perspectives regarding electronic informed consent

Two researchers (EDS and DZ) carried out data analysis together using Excel. A combination of deductive and inductive thematic analysis of the ethical, legal, regulatory, and user interface perspectives was used, reporting different concepts and the main findings associated with them. Thematic analysis was conducted according to the six-phase approach described by Braun and Clarke [19]. During the first phase, notes were created on the ethical, legal, regulatory, or user interface perspectives found in literature. These notes were valuable for the creation of initial codes in the second step. During the third step, we clustered these codes to generate broad concepts. Thereafter, the concepts were thoroughly reviewed and were defined in the fourth and fifth step. In the sixth and last step of this approach, we provided a descriptive overview to summarize the concepts found in literature [19]. Within these concepts, studies reporting similar findings were grouped together in order to provide a concise overview of results.

### Quality Assessment

Two researchers (EDS and DZ) assessed the quality of all included studies. Based on the study design, the Critical Appraisal Skills Programme (CASP) for qualitative studies [20], Mixed Methods Appraisal Tool (MMAT) for mixed methods studies [21], and Jadad tool for randomized controlled trials [22] were used. MMAT and CASP were used to criticize study aspects such as the aim and the methodology of the study [20,21]. These aspects were evaluated using a categorical scale (yes, indeterminate, or no), and thereafter, we converted the number of positive assessments into percentages. The quality of randomized controlled trials was assessed by considering the randomization, blinding, dropouts, and withdrawals. Randomized controlled trials could receive up to 5 points using the Jadad tool [22]. The Newcastle-Ottawa scale for cohort studies and an adapted version of this scale for cross-sectional studies were applied. Using the Newcastle-Ottawa scale, cohort and cross-sectional studies were evaluated using the following quality parameters: selection of study groups, comparability of study groups, and ascertainment of the outcome. Moreover, this scale also assessed the statistical test used in cross-sectional studies. Scores ranged from 0 to 9 for cohort studies and from 0 to 10 for cross-sectional studies [23,24].

## Results

### Search Results

**Figure 1:**
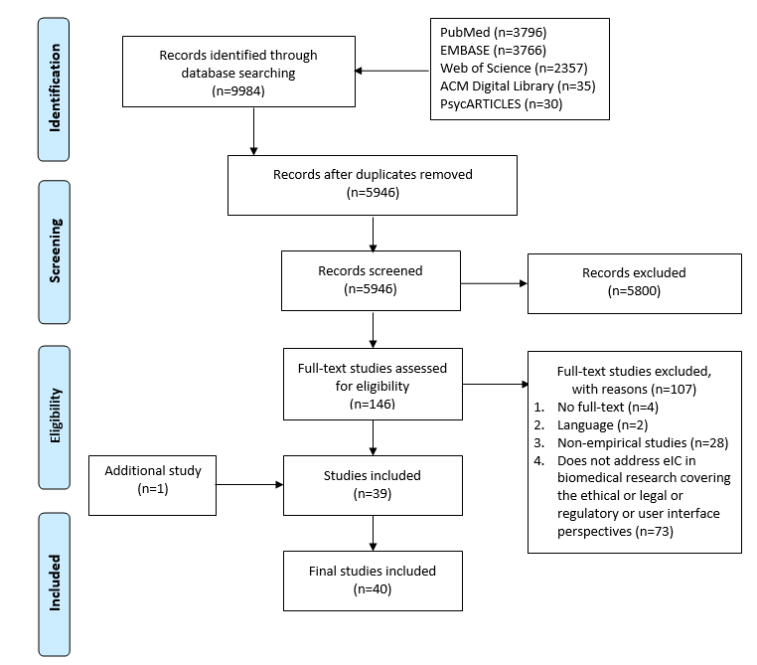
PRISMA flowchart of the systematic review. eIC: electronic informed consent.

Our search strategy produced a total of 9984 studies. After the screening process (Figure 1), 40 studies were included in the final analysis.

### Characteristics of Included Literature

Multimedia Appendix 2 summarizes the main characteristics for each study. The majority of studies were conducted in the United States (27 studies), followed by the United Kingdom (3 studies). There was 1 study each in Ireland, Korea, Canada, Australia, Spain, Germany, and Denmark; 3 studies reported results from several countries. The designs of the studies included 10 randomized controlled trials, 10 mixed methods studies, 10 cross-sectional studies, 9 qualitative studies, and 1 cohort study. Most stakeholder groups involved in the studies were represented by research participants (26 studies), followed by researchers (2 studies), and ethics committee members (1 study). In 11 studies, more than 1 of these categories, including health authorities, were involved. As for the characteristics of the research participants, 22 studies included different age categories (range 18-88 years), 3 studies each were conducted in young and elderly populations, and only 1 study was conducted in children. No information regarding age was reported in 5 studies involving research participants. When the information was provided, we noticed that, in 12 studies, the majority of research participants had a college degree or more, in 8 studies the majority of research participants had less than a college degree, and in 2 studies there was an equal distribution in education level of the research participants. Meanwhile, in 12 studies there was no information regarding the education level of the research participants. Studies were conducted in both female and male populations, except 4 studies that only included either women or men. The tools used to collect relevant information varied, based, also, on the study design. Multimedia Appendix 3 shows an overview of the intervention, scenario, and tools used.

### Quality Assessment

The quality of the included studies varied widely. Among the randomized controlled trials, only 3 studies [25-27] received a quality score of 3. Quality scores of 2 and 1 were provided to 1 study [28] and 6 studies [29-34], respectively. The qualitative studies ranged from satisfying 80% or more of the quality criteria (6 studies) [35-40] to satisfying 50% to 60% of the quality criteria (3 studies) [41-43]. Moreover, 3 mixed methods studies met 80% or more of the quality criteria [16,44,45], 6 studies met from 57% to 71% of the quality criteria [15,46-50], while 1 met only 43% of the quality criteria [51]. The only cohort study had a quality score of 7 [52], while the quality scores of cross-sectional studies were assessed to be 4 [53-56], 5 [57-61], and 6 [62].

### Domain Perspectives

Perspectives of the ethical, legal, regulatory, and user interface domains were reported using 6 concepts: format, impact on understanding, acceptability, security and trust, storage, and content. Due to the cross-disciplinary nature of these perspectives, they were not reported for each domain individually as each of these concepts touches on 2 or more domains.

### Format

A range of aspects related to the format were identified in the literature review. The majority of research participants, researchers, members of the ethics committee as well as health authorities believed that the ability to incorporate audio, video, or graphics in electronic informed consent is a distinct benefit, especially for vulnerable groups [15,16,28,33,39,43,45, 47-49,57]. Patients who were older adults, for example, expressed the usefulness of graphics and audio [45]. Research participants mentioned in a focus group that video and graphics may be more effective than written text in conveying information [16]. Moreover, patients involved in a study of Simon et al [39] indicated that audio narration could be of help for research participants with poor eyesight or limited literacy. However, participants argued for caution because background music and sound effects could be an added distraction [50]. In contrast, video was considered a multimedia element that might hold participants’ attention more than a paper-based informed consent could [33]. Important to consider is the length of the video, since research participants highlighted that a 5-minute video was too long [33]. The use of graphics, including icons, was appreciated by all involved stakeholder groups [15,16,28,39,43,45,47-49,57]. For instance, the implementation of a progress bar was advised by researchers to indicate the different steps of the electronic informed consent form [43]. A pilot study [48] involving children highlighted the entertainment of graphics, making the electronic informed consent platform a pleasure to use. On the contrary, adult patients with fragile X syndrome, a genetic disorder causing intellectual impairment, had difficulty understanding aspects of clinical research such as blinding and randomization explained by several animations [47].

Furthermore, hyperlinks were identified as an important aspect of the format by participants and researchers [16,43,56]. Hyperlinks could be used as a video link between researchers and participants in order to combine an online with a face-to-face consent process [56]. Moreover, patients engaged in focus groups elicited hyperlinks as an encouraging way to seek additional information [16]. Nevertheless, it should be noted that only 20 out of 491 patients (4.1%) involved in a randomized controlled trial clicked on 1 or more hyperlinks [26]. Members of the ethics committee and health authorities advised avoiding the use of hyperlinks to webpages that may modify their content [15].

Researchers and ethics committee members as well as research participants criticized having extensive content during usability analysis of electronic informed consent platforms [34,54]. Research participants advised using a bullet point format with access to additional information if desired [28,34,50]. Simple, concise language should be used to encourage a sufficient level of understanding [15,28,40,50]. The possibility of marking information that participants did not understand was considered useful and was viewed as an opportunity to enhance the discussion with the research staff [15]. Nonetheless, research participants and researchers emphasized not losing the personal connection between researcher and participant [15,50].

Moreover, electronic informed consent offers the possibility to give information in several languages by using subtitles or translated text. Although many factors influence participant recruitment, researchers and research participants believed that electronic informed consent may facilitate recruitment, particularly in rare disease research [35]. By translating the content of electronic informed consent into different languages, information is available for a large number of individuals regardless of their geographical setting [35,53].

Little information was provided about the choice of the device when using an electronic informed consent platform. Two studies, both of them using touchscreen formats, reported incorrect end user input [51,53]. Researchers entered incorrect medical record numbers and patient names containing spelling errors [53]. A similar case with research participants was reported; in this particular study [51], several participants accidentally removed their signature, which contributed to the majority of errors when using touchscreen devices. Moreover, research participants who were older adults reported that they would need training to be able to use a tablet-based consent process [45].

### Impact on Understanding

Electronic informed consent may have an impact on the understanding of research participants by implementing a quiz, before signing the consent, to assess the participants’ level of comprehension [28,35,39,44,55]. However, research participants argue for caution (to not make it feel like a test) [50]. In addition, interactive technology and a printed consent form offer the opportunity to review information giving participants the time to learn about the research [15,16,37,39,50]. Patients considered a paper-based form more permanent and suggested implementing a printout option in the electronic informed consent technology [39]. In addition, Vanaken et al [15] reported that some research participants preferred to take a printed version of the informed consent document home. Hence, the pressure to give consent immediately was decreased [16]. An extensive amount of studies investigated barriers related to the use of electronic informed consent which could prevent research participants from achieving a reasonable level of understanding [15,25-28,31-34,39,40,44,45,48,49,52,53,55,60,61]. For the majority of studies, no barriers were reported [25-28,31-34,44,48,52]. For example, patients with mental disorders, of whom the majority had a primary level of education, were able to make well-informed decisions by using electronic informed consent [52]. Moreover, patients with schizophrenia had better understanding of disclosed information when using electronic informed consent compared to when using paper-based informed consent [32]. In a few studies, barriers impeding adequate comprehension were reported by research participants, researchers as well as ethics committee members regarding people with limited computer literacy, visual or auditory impairment, and regarding the lack of access to computers or internet [15,39,40,45,53,60,61]. Other reported barriers to achieving adequate understanding were the attitudes of research participants looking for additional information after reading a very concise informed consent and the use of graphics that may be unclear [47,49,55].

### Acceptability

Some researchers had concerns regarding approval of electronic informed consent as an alternative consent process by ethics committees [16,61]. In addition, ethics committees themselves expressed uncertainty about the impact of the audio-visual aspects on the ethical review process and the review duration [15,59,61]. The ability to confirm the identity of research participants consenting remotely may be challenging, especially for researchers [57]. In general, varying perceptions were reported regarding the use of electronic signatures. Although the majority of ethics committee chairpersons enrolled in a study by Kane et al [59] did not encounter submissions of informed consent documents containing electronic signatures, a large number of involved chairs would approve it. However, researchers and members of ethics committees were unsure about compliance with local regulations [15,59]. Some representatives of health authorities supported the use of electronic signatures while others were concerned about data privacy [15]. With respect to the research participants, a study by Haussen et al [60], involving legal authorized representatives of whom 21 out of 53 representatives (40%) had a low educational status, stated that for these participants there is an increased chance of preferring a paper-based informed consent.

### Security and Trust

Research participants stressed the importance of trust in the authenticity of electronic informed consent to share health data and to agree to take part in the study [36]. This went hand-in-hand with security of the electronic informed consent platform, which was a main concern for all stakeholders [15,34,36,39,40,42,50,61]. A secure platform may enable the transfer of files, which researchers considered an important factor in biomedical research [57]. Chhin et al [53] reported that the electronic informed consent platform that they developed could only be accessed by using individual user accounts and passwords. Moreover, researchers mentioned the need for providing sufficient information on privacy aspects to potential research participants [43]. According to research participants, electronic informed consent may enhance trust in research because of the possibility of returning research information by using innovative technology [38]. Nevertheless, Harle et al [26] conducted a randomized controlled trial with patients receiving standard, interactive-only, or interactive trust-enhanced electronic informed consent. Trust-enhanced messages were implemented containing additional information on data protections, regulations, and training of the research staff. They indicated there was no effect from the inclusion of these trust-enhanced messages on data sharing, satisfaction, and understanding [26].

### Storage

According to multiple stakeholders, electronic storage of consent details constitutes a notable benefit by enabling researchers to have a trustworthy and traceable overview of the consent status of participants [15,35]. For example, electronic informed consent can support online withdrawal, together with documentation of the reasons for withdrawal [41,46]. Moreover, online storage improves version control which might reduce the number of adverse inspection findings by the health authorities. Nevertheless, health authorities voiced concerns that informed consent forms may be inaccessible during an inspection [15]. An important feature for researchers and the ethics committee is to have the ability to control access for specific consent documents [57]. According to research participants, access to their own health information is a key benefit of participation [46]. Research participants requested more transparency regarding the use of their data [38,58]. They expressed the right to control the sharing and use of their private health information [37,42]. Moreover, electronic informed consent enables researchers to update participants frequently with information about preliminary results, follow-up studies, and main outcomes [35]. Nevertheless, the majority of patients with mental disorders, involved in a study by Sundby et al [62], stated that they would prefer direct contact with the research staff for receiving genomic information concerning serious or life-threatening conditions.

### Content

The usefulness of additional content elements such as definitions was articulated in a focus group involving research participants who were older adults [45]. Moreover, exposing research participants to social annotation, such as comments generated by end users on several aspects of the electronic informed consent form, was considered important to feel adequately informed [29]. However, the emotional force communicated in social annotations has an influence on research participants’ perceptions with regard to information given in electronic informed consent. Research participants exposed to positive valence annotations indicated feeling less informed than participants receiving a combination of positive and negative valence annotations [30]. Various studies [15,37,58,62] provided insights into a personalized approach of an electronic informed consent platform. Research participants stated that they would like to receive personalized elements and tailored information such as the display of their name in the electronic informed consent form or the impact of their contribution on a specific research question [15,37]. Moreover, Kim et al [58] conducted a study using an electronic informed consent platform in which research participants were allowed to modify their preferences for data sharing. In this study [58], research participants indicated that a personalized approach could enable participants’ eagerness for data sharing for research purposes. Furthermore, researchers appreciated the possibility of research participants indicating what kind of information they would like to obtain [62].

## Discussion

### Importance of Understanding in Electronic Informed Consent

The majority of studies paid particular attention to understanding, considering that it is seen as a crucial point in enabling participants to give their informed consent. In some studies [28,33,34,39,44-46,48,55,60,62], the majority of participants had a high level of education. It should also be noted that a number of studies [27,29,51] assessing comprehension did not report the education level of their participants. Therefore, there might be additional barriers for less educated participants to achieving an adequate level of understanding. If research participants are not adequately informed about a research study, they may be disappointed due to misconceptions of the benefits. As a result, researchers may face increased dropout. Moreover, research participants may distrust ethics committees when they do not put the research participant at center when reviewing an electronic informed consent. Literature reported that electronic informed consent could improve understanding through the opportunity to check participants’ level of comprehension by using quizzes before electronically signing the consent form [28,35,39,44,55]. The implementation of a quiz may prevent research participants from immediately agreeing to participate in research, not allowing the content to be reviewed thoroughly. Nevertheless, meta-analysis of different informed consent interventions conducted by Nishimura et al [10] showed a significant higher understanding for paper-based enhanced consent forms including simplified text and facilitated reading level. A non-significant increase of understanding was observed for multimedia interventions. These enhanced consent forms and multimedia approaches were compared with a control consent process that consisted of a paper-based informed consent or an already enhanced informed consent [10]. It would, therefore, seem that further research is needed to explore the effect on understanding of an electronic informed consent platform including all of these aspects, considering the education level, age, health status, and health literacy of the participant.

### Particular Attention to Specific Population Groups

Several studies [15,39,40,45,60,61] included in this review reported concerns about access to electronic informed consent for specific population groups. From an ethical point of view, different population groups need to have the opportunity to be represented in biomedical research. The possibility for several population groups to take part in a research study may, first, broaden their access to treatments, and second, positively impact the generalizability of research results. Adequate support is required for participants with, for example, no or limited computer literacy. Obtaining informed consent in people with mental disorders remains a challenge that may be overcome with electronic informed consent. Health authorities believe that vulnerable populations, which are often underrepresented in clinical research, might benefit [15]. However, it is important to highlight that only a limited number of studies [25,31,32,45,47,48,62] included vulnerable groups who may have particular requirements. In only 1 pilot study, perceptions of children regarding the use of graphics were evaluated [48]. Recommendations cannot be inferred from this pilot study because they require verification in further research. Generally, electronic informed consent platforms are intended to be used by multiple dissimilar target groups. The target population varies in literacy, education, age, health condition, and many other factors. Therefore, preferences for designing a usable interface may deviate across the type of end users. Visual factors, such as the font size or the use of graphics, will differ for research studies involving older adults, children, or visually impaired participants.

### Personal Connection

Attention needs to be paid to not losing the personal connection between research participants and research staff. Electronic informed consent has the opportunity to supplement the existing paper-based consent process but is not meant to replace it. For example, research participants may prefer the paper-based consent document or electronic informed consent facilitated by a discussion with the research staff to enable an informed decision. To enhance trust in research or in the authenticity of an electronic informed consent platform, the personal connection may play an important role. Moreover, electronic informed consent has the potential to inform research participants about early findings, but participants involved in a study by Sundby et al [62] preferred having direct contact with the research team if this information was related to life-threatening conditions. In addition, the online consent form should provide participants with a link that they can enter to withdraw their consent any time they desire. Nevertheless, for several types of medication, it is dangerous to abruptly stop [63]. For this reason, the interaction between the research participant and the research team is of utmost importance to prevent withdrawal symptoms by giving all necessary information. In general, a lack of face-to-face communication may lead to misunderstanding with regard to several aspects of a research study.

### Guidance Framework

Acceptability of electronic informed consent by the health authorities was unclear [15,59]. Therefore, analysis of the legal framework regarding different aspects of electronic informed consent is required to determine the restrictions in certain jurisdictions. More research is needed to explore the legal aspects related to electronic informed consent across several countries. In addition, uncertainty exists about the review process of electronic informed consent by ethics committees [15,59,61]. Due to the lack of experience, it remains unclear how the evaluation process of ethics committees will be impacted. It would thus be of interest to investigate which aspects of the electronic informed consent process ethics committees would consider for evaluation. General consensus on the review process of ethics committees is required to facilitate harmonized review for multicentric studies in which multiple ethics committees are involved. In addition, researchers need to receive clarification on which materials they need to submit to ethics committees. A guidance framework could reduce the burden of researchers and ethics committees concerning the preparation and review of electronic informed consent and could ensure protection of the rights, safety, and welfare of human participants.

### Privacy by Design

Notable concerns were expressed by several stakeholders regarding security of electronic informed consent platforms, and thus data privacy [15,34,36,39,40,42,50,61]. Because electronic informed consent establishes the opportunity to give consent remotely, capture of an electronic signature and proof of identity are challenging. Researchers and ethics committees raised concerns about low compliance of electronic signature with local regulations. These concerns may strengthen reluctance to implement and use electronic informed consent platforms in biomedical research. In order to secure the platform, potential security threats need to be identified to counter them in security software. It is of utmost importance to implement the highest standards in security and privacy design to prevent fraudulent use of participants’ health data. Moreover, clarifying the integrated security and privacy aspects to the research participants is valuable to reduce these concerns. A breach of privacy could, for research participants, lead to job loss or consequences related to their insurance. Moreover, researchers could be held responsible for misconduct. Ethics committees and health authorities could also be held responsible, as they did not look into the security of the electronic informed consent platform when reviewing or inspecting. In previous years, the failure to obtain and document the consent of research participants was part of frequent FDA inspection failures [64]. Owing to online storage, electronic informed consent may contribute to, for example, better version control and documentation of the consent process [15,35]. Nevertheless, controlled access systems need to be implemented to restrict access for stakeholders for different types of content in order to respect the privacy of the participants.

### Usability

Noteworthy is the discrepancy between quantitative and qualitative usability testing. Despite the mention of hyperlinks as an important feature of the user interface in a qualitative focus group study [16], this feature was barely used in a randomized controlled trial evaluating different electronic informed consent platforms [26]. These results suggest using an iterative design cycle starting with an evaluation of what participants would consider as useful in the user interface, after which a usability analysis should be performed. It is crucial to involve research participants when designing an electronic informed consent platform to create a user-friendly and acceptable system. This will support researchers in ensuring an adequate number of participants in order to detect possible important clinical findings. Moreover, although research participants indicated preferring concise information in consent forms [15,28,40,50], attention needs to be paid to participants who are not motivated to seek additional information. Specific measures are needed if input of researchers or research participants is required when using electronic informed consent in order to prevent errors. Potential errors are related to devices used by biomedical research stakeholders and the amount of manual input required. The use of touchscreen devices might invoke more inadvertent actions from accidental touches on the display, compared to those from the use of computers. The implementation of a quality assurance procedure is highly recommended in order to avoid incorrect end-user input.

### Inclusivity

Language aspects may complicate obtaining informed consent, and thus recruitment. It requires additional effort to involve research participants whose primary language is not frequently used in the informed consent process. To make the informed consent process more inclusive, subtitles or translated text can be used in electronic informed consent. Additionally, intercultural mediation can be considered to reduce the adverse outcomes of language barriers. An intercultural mediator can be part of the electronic informed consent process to assist the linguistic interpretation of information in the electronic informed consent [65].

### Toward Personalized Electronic Informed Consent

An electronic informed consent platform provides an important means for modifying consent preferences. Electronic informed consent can be tailored to participants’ needs to address their preferences. In this way, participants may change different aspects such as which information they would like to receive, how they prefer to be contacted, and how often they wish to be contacted. Research participants indicated that access to their personal health data is an important motivation to participate in research studies. By enabling a personalized approach in the electronic informed consent platform, research participants can indicate whether they would like to receive this information. Moreover, electronic informed consent can be personalized for different kind of diseases. Patient preferences can vary for disease-specific informed consents. Participants with delicate health conditions may, for example, require additional information related to the study and may not be eager to share their data. Personalization may improve research participants’ engagement and understanding. However, attention needs to be paid to the review process of such personalized electronic informed consent in order to avoid a time-consuming process. Further research that considers several types of end users is recommended, as electronic informed consent must be tailored to different population needs. Moreover, electronic informed consent provides the possibility of updating research participants with information on an ongoing research study. Nevertheless, it needs to be investigated how an electronic informed consent platform can be personalized and how longitudinal interaction can be assured.

### Recommendations

The ethical, legal, regulatory, and user interface perspectives were converted into recommendations to facilitate implementation of electronic informed consent in biomedical research. The recommendations are based upon the 6 concepts reported in the results and are shown in Table 1.

**Table 1:** Recommendations to guide implementation of electronic informed consent in biomedical research.

Concept	Description
Format	Implement audio, video, graphics (ie, icons, progress bar) and hyperlinks. Note: do not add audio that may distract participants, consider the length of the video, create understandable graphics and avoid hyperlinks to webpages with dynamic content Use simple, concise language and implement a bullet point format. Note: provide access to additional information if desired Give the possibility to research participants to highlight information that is difficult to understand in order to facilitate the discussion with the research staff Depending on the research study, make electronic informed consent available in multiple languages by using subtitles or translated text Implement a quality assurance process to check the input of the end user
Impact on understanding	Pay attention to the personal connection between the research participants and the research staff (also refers to security and trust) Implement quizzes to assess the participants’ level of comprehension. Note: do not let the quizzes feel like an evaluation Give the possibility to review information by using interactive technology or the printed electronic informed consent form Guarantee adequate support for people with limited computer literacy, visual/auditory impairment and people who do not have access to internet or computers
Acceptability	Collaborate with health authorities and ethics committees to create a framework for reviewing and implementing electronic informed consent
Security and trust	Implement controlled access systems for several stakeholder groups and pay attention to a secured electronic informed consent platform. Note: provide sufficient information to potential research participants about privacy aspects of the platform Make sure that the secure transfer of files is possible between stakeholder groups
Storage	Provide online storage of the informed consent Support online withdrawal with documentation of the reasons for withdrawal Pay attention to transparency regarding the use of participants’ health information and their right to control the sharing and use of this information Implement the possibility to update research participants frequently with information about preliminary results, follow-up studies and main outcomes
Content	Implement definitions Implement social annotations but mind the emotional force Implement a personalized approach (eg, by letting the participants indicate what kind of information they would like to receive)

### Limitations

Our systematic review only included literature published in English. As a consequence, selection bias could have been introduced because it is possible that information specific to the ethical, legal, regulatory, or user interface domain of electronic informed consent in languages other than English was not identified. Another limitation was that the methodological quality of the included studies was rated overall as moderate which needs to be considered when interpreting the reported findings. Most of the studies had small sample sizes, which could be an issue for the generalizability of results. The studies included in the review had a high level of heterogeneity among them, which is the reason why a descriptive analysis was conducted. The differences in study designs, research fields and contexts, populations, and tools used to assess the results may have impacted the findings reported by each study.

### Conclusions

This systematic review highlights different opportunities and challenges for responsible implementation of electronic informed consent in biomedical research. Electronic informed consent provides the possibility of enforcing a personalized approach and supporting a longitudinal interaction with research participants. Findings suggest that electronic informed consent may have a beneficial impact on the consent process as long as some requirements are fulfilled. Special attention needs to be paid to specific population groups and to personal interaction with research staff. Future high-quality research, especially using randomized controlled trials, is required to provide information that may encourage the use of electronic informed consent for vulnerable groups.

## Appendix

Multimedia Appendix 1 Search strategy.

Multimedia Appendix 2 Main characteristics of the 40 included studies.

Multimedia Appendix 3 Intervention, scenario, and tools of the 40 included studies.

## Abbreviations

CASP: Critical Appraisal Skills Programme
FDA: US Food and Drug Administration
MMAT: Mixed Methods Appraisal Tool
PRISMA: Preferred Reporting Items for Systematic Reviews and Meta-Analyses
