# Supplementary material for: Implementation of Electronic Informed Consent in Biomedical Research and Stakeholders’ Perspectives: Systematic Review
Source: J Med Internet Res. 2020 Oct 8;22(10):e19129. doi: 10.2196/19129 (PMC7582148; doi:10.2196/19129)
Supplement: Multimedia Appendix 1 [file jmir_v22i10e19129_app1.doc]

PubMed:

(online[tiab] AND informed-consent*[tiab]) OR “online consent”[tiab] OR “online consenting”[tiab] OR “electronic informed consent”[tiab] OR “electronic consent”[tiab] OR “electronic consenting”[tiab] OR “dynamic informed consent”[tiab] OR “dynamic consent”[tiab] OR “dynamic consenting”[tiab] OR e-consent*[tiab] OR econsent[tiab] OR econsenting[tiab] OR ((consent*[tiab]) AND (digital[tiab] OR interactive[tiab] OR multimedia[tiab] OR multimedia[MesH] OR video[tiab] OR telecommunications[MesH] OR telecommunication*[tiab] OR computer*[tiab] OR computers[MesH] OR “user-computer interface”[MesH] OR "online systems"[Mesh:NoExp] OR telemedicine[MesH] OR telemedicine[tiab])) OR ((“informed consent”[MesH]) AND (online[tiab] OR electronic[tiab] OR dynamic[tiab] OR digital[tiab] OR interactive[tiab] OR multimedia[tiab] OR multimedia[MesH] OR video[tiab] OR telecommunications[MesH] OR telecommunication*[tiab] OR computer*[tiab] OR computers[MesH] OR “user-computer interface”[MesH] OR "online Systems"[Mesh:NoExp] OR telemedicine[MesH] OR telemedicine[tiab])) AND ("2010/01/01"[Date - Publication] : "2019/11/14"[Date - Publication]) AND English[Language]

EMBASE:

'digital informed consent':ti,ab,kw OR 'digital consent*':ti,ab,kw OR 'online informed consent':ti,ab,kw OR 'online consent*':ti,ab,kw OR 'electronic informed consent':ti,ab,kw OR 'electronic consent*':ti,ab,kw OR 'dynamic informed consent':ti,ab,kw OR 'dynamic consent*':ti,ab,kw OR 'interactive informed consent':ti,ab,kw OR 'interactive consent*':ti,ab,kw OR 'e consent*':ti,ab,kw OR econsent*:ti,ab,kw OR ((consent*:ti,ab,kw) AND (multimedia:ti,ab,kw OR multimedia/exp OR video:ti,ab,kw OR telecommunication/exp OR telecommunication*:ti,ab,kw OR 'information technology device'/exp OR computer*:ti,ab,kw OR 'online system'/exp OR telemedicine/exp OR telemedicine:ti,ab,kw)) OR (('informed consent'/exp) AND (online:ti,ab,kw OR electronic:ti,ab,kw OR dynamic:ti,ab,kw OR digital:ti,ab,kw OR interactive:ti,ab,kw OR multimedia:ti,ab,kw OR multimedia/exp OR video:ti,ab,kw OR telecommunication/exp OR telecommunication*:ti,ab,kw OR 'information technology device'/exp OR computer*:ti,ab,kw OR 'online system'/exp OR telemedicine/exp OR telemedicine:ti,ab,kw)) NOT 'conference abstract':it AND [english]/lim AND [2010-2019]/py

Web of Science:

TS=("digital informed consent" OR "digital consent*" OR "online informed consent" OR "online consent*" OR "electronic informed consent" OR "electronic consent*" OR "dynamic informed consent" OR "dynamic consent*" OR "interactive informed consent" OR "interactive consent*" OR "e consent*" OR econsent* OR ((consent*) AND (multimedia OR video OR telecommunication* OR computer* OR telemedicine))) AND PY=(2010-2019)

ACM Digital Library:

recordAbstract:"digital informed consent" OR "digital consent*" OR "online informed consent" OR "online consent*" OR "electronic informed consent" OR "electronic consent*" OR "dynamic informed consent" OR "dynamic consent*" OR "interactive informed consent" OR "interactive consent*" OR e-consent* OR econsent* OR ((consent*) AND (multimedia OR video OR telecommunication* OR computer* OR telemedicine))

PsycARTICLES:

AB,TI("digital informed consent" OR "digital consent*" OR "online informed consent" OR "online consent*" OR "electronic informed consent" OR "electronic consent*" OR "dynamic informed consent" OR "dynamic consent*" OR "interactive informed consent" OR "interactive consent*" OR "e consent*" OR econsent*) OR ((AB,TI(consent*)) AND (MAINSUBJECT.EXACT.EXPLODE("telemedicine" OR "multimedia" OR "telecommunications media") OR AB,TI(multimedia OR video OR telecommunication* OR computer* OR telemedicine))) OR ((MAINSUBJECT.EXACT.EXPLODE("informed Consent")) AND (AB,TI(digital OR online OR electronic OR dynamic OR interactive OR multimedia OR video OR telecommunication* OR computer* OR telemedicine) OR MAINSUBJECT.EXACT.EXPLODE("telemedicine" OR "multimedia" OR "telecommunications Media"))) AND YR(>=2010)
