# Supplementary material for: Implementation of Electronic Informed Consent in Biomedical Research and Stakeholders’ Perspectives: Systematic Review
Source: J Med Internet Res. 2020 Oct 8;22(10):e19129. doi: 10.2196/19129 (PMC7582148; doi:10.2196/19129)
Supplement: Multimedia Appendix 2 [file jmir_v22i10e19129_app2.docx]

Main characteristics of the 40 included studies.

| **Author** | **Study design** | **Location** | **Target population** | **Age** | **Sex** | **Education level** | **Sample size** |
| --- | --- | --- | --- | --- | --- | --- | --- |
| Abujarad et al [44] | ● Mixed methods | ● United States | ● Focus group: research participants (patients with asthma), researchers, EC member  ● Usability evaluation: research participants (patients with asthma) | ● Focus group: N/A ● Usability evaluation: age range (years): 21-74 | ● Focus group: N/A ● Usability evaluation: 55.6% women | ● Focus group: N/A ● Usability evaluation: 11.1% high school graduate, 11.1% at least some college, 77.8% at least bachelor's degree | ● Focus group: patients: n=2, researchers: n=2, EC member: n=1 ● Usability evaluation: n=9 |
| Anderson et al [16] | ● Mixed methods | ● United States | ● Research participants: (patients) with research experience  ● Researchers | ● Research participants: age range (years): 26-60 (63.6% of participants) and 36.4% of participants >60 years ● Researchers: age range (years): 26-60 (78.9% of researchers) and 21.1% of researchers >60 years | ● Research participants: 77.3% women ● Researchers: 73.7% women | ● Research participants: 27.3% less than high school diploma, 13.6% high school or GED, 4.5% technical college/trade school diploma, 31.8% some college, 9.1% bachelor's degree, 13.6% master's degree ● Researchers: 5.3% technical college/trade school diploma, 15.8% bachelor's degree, 31.6% master's degree, 37.4% doctoral degree or professional degree | ● Patients: n=22 ● Researchers: n=19 |
| Balestra et al [29] | ● RCT | ● United States | ● Research participants | ● Mean age (years): 35.5 (SD=10.7) | ● 58.8% women | ● N/A | ● n=259 |
| Balestra et al [30] | ● RCT | ● United States | ● Research participants | ● Mean age (years): 34.25 (SD=10.78) | ● 48.3% women | ● Some high-school education (n=1), high school diploma (n=12), some college education (n=58), bachelor’s degree (n=59), master’s degree (n=14), doctoral degree (n=3) and unknown (n=2) | ● n=149 |
| Bobb et al [27] | ● RCT | ● United States | ● Research participants (patients) | ● Mean age (years): 54.4 (SD=16.9) | ● 45% men | ● N/A | ● n=100 |
| Budin-Ljøsne et al [35] | ● Qualitative | ● European countries | ● Researchers  ● Research participants  ● EC members | ● N/A | ● N/A | ● N/A | ● N/A |
| Bunnell et al [57] | ● Cross-sectional | ● United States | ● Researchers  ● EC members | ● N/A | ● N/A | ● N/A | ● n=134 |
| Chen et al [41] | ● Qualitative | ● United States | ● Research participants (patients) | ● N/A | ● N/A | ● N/A | ● n=434 |
| Chhin et al [53] | ● Cross-sectional | ● Canada | ● Researchers | ● N/A | ● N/A | ● N/A | ● Feasibility study: n=86  ● User survey: n=71 |
| Doerr et al [46] | ● Mixed methods | ● United States | ●Research participants (participants with and without Parkinson disease) | ● Mean age (years): 35.9 | ● 21.55% women | ● 57.53% ≥4-year college degree | ● n=9846 |
| Furberg et al [47] | ● Mixed methods | ● United States | ● Research participants (patients with a diagnosis of FXS) ● Researchers | ● Research participants (interview): mean age (years): 22.3 | ● Research participants (interview): 100% men | ● N/A | ● Research participants:  survey: n=104, interview: n=9 ● Researchers: n=3 |
| Ham et al [31] | ● RCT | ● Korea | ● Research participants (patients with benign prostatic hyperplasia) | ● Mean age (years): 66.9 (SD=6.4) | ● 100% men | ● 37.5% middle school or less, 32.5% high school, 30% university or more | ● n=40 |
| Harle et al [40] | ● Qualitative | ● United States | ● Research participants | ● Mean age (years): 54.1 (SD=14.3) | ● 87.5% women | ● 31.3% high school or less, 25.0% some college, 43.8% master’s degree or higher | ● n=32 |
| Harle et al [26] | ● RCT | ● United States | ● Research participants (patients) | ● Mean age (years): 45.6 (SD=16.0) | ● 68.4% women | ● 9.8% less than high school, 33.9% high school graduate or GED, 31.9% some college, 14.4% bachelor’s degree, 9.9% master’s, professional, or doctorate degree | ● n=734 |
| Harmell et al [32] | ● RCT | ● United States | ● Research participants (outpatients with schizophrenia and normal comparison subjects) | ● Normal comparison subjects: mean age (years): 50.6 (SD=14.0)  ● Schizophrenia group: mean age (years): 57.5 (SD=9.42) | ● Normal comparison subjects: 56.3% women  ● Schizophrenia group: 26.3% women | ● Normal comparison subjects: mean years education: 15.5 (SD=1.8)  ● Schizophrenia group: mean years education: 12.1 (SD=1.4) | ● Normal comparison subjects: n=16  ● Schizophrenia patients: n=19 |
| Haussen et al [60] | ● Cross-sectional | ● United States | ● Legal authorized representatives | ● Age range (years): 28-59 | ● 64% women | ● 40% less than tertiary, 60% tertiary or more | ● n=53 |
| Iwaya et al [43] | ● Qualitative | ● Australia | ● Researchers | ● N/A | ● N/A | ● N/A | ● Usability evaluation: n=10 ● Interview: n=9 |
| Jayasinghe et al [45] | ● Mixed methods | ● United States | ● Research participants | ● Focus group: mean age (years): 77.47 (SD=7.54)  ● Randomized pilot: mean age (years): 74.65 (SD=7.36) | ● Focus group: 80% women  ● Randomized pilot: 85% women | ● Focus groups: 13% high school/GED, 20% some college, 47% college graduate, 20% postgraduate  ● Randomized pilot: 5% elementary, 10% high school/GED, 15% some college, 20% college graduate, 50% postgraduate | ● Focus group: n=15 ● Randomized pilot: n=20 |
| Kane et al [59] | ● Cross-sectional | ● United States | ● EC chairpersons | ● N/A | ● N/A | ● N/A | ● n=114 |
| Kim et al [58] | ● Cross-sectional | ● United States | ● Research participants (patients with chronic diseases) | ● N/A | ● 26% women, 2.4% not answered | ● 19.8% graduate level, 18.3% 4-year college, 27.8% high school-some college, 10.3% <high school, 23.8% not answered | ● n=126 |
| Madathil et al [51] | ● Mixed methods | ● United States | ● Research participants (patients) ● Researchers | ● Patients: age range (years): 18-77  ● Researchers: age range (years): 23-74 | ● N/A | ● N/A | ● Patients: n=40 ● Researchers: n=10 |
| Mahnke et al [50] | ● Mixed methods | ● United States | ● Researchers ● Research participants | ● Usability evaluation: age range (years): 48-78 | ● Usability evaluation: 67% women | ● N/A | ● Simulation of consents: research participants: n=6, researchers: n=5 ● Community advisory group focus groups: research participants: n=11 ● Hybrid focus groups: research participants: n=10 ● Usability evaluation: research participants: n=9 |
| McGowan et al [56] | ● Cross-sectional | ● United Kingdom  ● Ireland | ● Research participants | ● N/A | ● N/A | ● N/A | ● n=111 |
| Moran-Sanchez et al [52] | ● Cohort | ● Spain | ● Research participants (patients with psychotic, mood and anxiety disorders) | ● Mean age (years): 41.3 (SD=10.2) | ● 47.7% women | ● 59% primary, 32% secondary, 9% university degree | ● n=88 |
| Perrault et al [55] | ● Cross-sectional | ● United States | ● Research participants (students) | ● Mean age (years): 19.89 (SD=1.54) | ● 57.8% women | ● University students | ● n=429 |
| Ramos et al [49] | ● Mixed  methods | ● United States | ● Research participants (patients with HIV) | ● Post-test design: mean age (years): 54.60 (SD=10.8) | ● Post-test design: 25% women | ● Post-test design: 20% some high school/no diploma, 10% high school diploma/GED, 15% some college/no degree, 5% trade/vocational school, 15% associate degree, 30% bachelor degree, 5% graduate/ professional degree | ● Icon selection: n=5 ● Post-test design: n=20 |
| Rothwell et al [33] | ● RCT | ● United States | ● Research participants (women with full-term pregnancies who gave birth with normal birth outcomes) | ● N/A | ● 100% women | ● 1.61% some high school, 8.06% high school diploma/GED, 17.74% some college, 9.68% associate’s degree, 41.94% bachelor’s degree, 20.97% graduate degree | ● n=62 |
| Rowan et al [42] | ● Qualitative | ● Ireland | ● Research participants (students) | ● Age range (years): 25-34 | ● 25% women | ● Graduate business students | ● n=24 |
| Rowbotham et al [34] | ● RCT | ● United States | ● EC members  ● Researchers ● Research participants (patients) | ● Research participants: age range (years): 18-80 | ● Research participants: 66% women | ● Research participants: 43% college degree, 30% advanced degree, 4% high school | ● EC members and researchers: n=14 ● Research participants: n=55 |
| Schneiderheinze et al [54] | ● Cross-sectional | ● Germany | ● Research participants (patients) | ● Mean age (years): 44.1 | ● N/A | ● N/A | ● n=47 |
| Simon et al [28] | ● RCT | ● United States | ● Research participants (patients) | ● Age range (years): 18-86 | ● 76% women | ● 79% at least a college degree or higher | ● n=200 |
| Simon et al [39] | ● Qualitative | ● United States | ● Research participants (patients) | ● Age range (years): 43-82 | ● 45% women | ● 61% at least a college degree | ● n=50 |
| Simon et al [61] | ● Cross-sectional | ● United States | ● Researchers  ● EC members | ● N/A | ● N/A | ● N/A | ● n=65 |
| Spencer et al [38] | ● Qualitative | ● United Kingdom | ● Research participants (patients with chronic rheumatic disease) | ● Mean age (years): 61 (SD=13) | ● 58% women | ● N/A | ● n=40 |
| Sundby et al [62] | ● Cross-sectional | ● Denmark | ● Research participants (patients with a mental disorder and healthy volunteers)  ● Researchers | ● Patients: mean age (years): 46.1 (SD=12.3) ● Healthy volunteers: mean age (years): 46.9 (SD=12.3) ● Researchers: mean age (years): 49.5 (SD=12.4) | ● Patients: 74.7% women ● Healthy volunteers: 49.8% women ● Researchers: 65.4% women | ● Patients: 5.0% none higher, 35.0% short higher, 30.0% medium higher, 24.2% long higher, 5.8% other  ● Healthy volunteers: 1.7% none higher, 29.4% short higher, 32.0% medium higher, 32.7% long higher, 4.1% other ● Researchers: 100% long higher | ● Patients: n=241 ● Healthy volunteers: n=2294 ● Researchers: n=102 |
| Tait et al [48] | ● Mixed methods | ● United States | ● Research participants (parents and children) | ● Parents: age range (years): 38-50 ● Children: age range (years): 8-14 | ● Parents: 40% women ● Children: 50% women | ● Parents: 20% high school, 80% college graduates | ● Parents: n=5 ● Children: n=4 |
| Teare et al [37] | ● Qualitative | ● United Kingdom | ● Research participants (patients and healthy volunteers) | ● N/A | ● N/A | ● N/A | ● n=32 |
| Vanaken et al [15] | ● Mixed methods | ● Argentina ● Australia  ● Canada  ● China  ● Denmark  ● Germany  ● Japan  ● Sweden  ● United Kingdom  ● United States | ● Research participants  ● Health authorities  ● EC members  ● Researchers | ● Research participants:  survey: age range (years): 18-64 and 27% of participants ≥65 years; Participant advisory board: age range (years): 25-87 | ● Research participants:  survey: 49% women; Participant advisory board: 60% women | ● N/A | ● Participant survey: n=3045 ● Participant advisory board: n=10 |
| Warriner et al [25] | ● RCT | ● United States | ● Research participants (patients with osteoporosis) | ● Mean age (years): 70.4 (SD=8.0) | ● 100% women | ● 6.06% less than high school, 27.27% high school or GED, 36.36% some college, 30.30% 4-year college or higher | ● n=33 |
| Wood et al [36] | ● Qualitative | ● United Kingdom | ● Research participants | ● Age range (years): 20-69 | ● 50% women | ● N/A | ● n=42 |

Abbreviations

EC: Ethics committee

FXS: Fragile X syndrome

GED: General educational development

N/A: Not applicable

RCT: Randomized controlled trial
