# Supplementary material for: Implementation of Electronic Informed Consent in Biomedical Research and Stakeholders’ Perspectives: Systematic Review
Source: J Med Internet Res. 2020 Oct 8;22(10):e19129. doi: 10.2196/19129 (PMC7582148; doi:10.2196/19129)
Supplement: Multimedia Appendix 3 [file jmir_v22i10e19129_app3.docx]

Intervention, scenario, and tools of the 40 included studies.

| **Author** | **Intervention** | **Scenario** | **Tool** |
| --- | --- | --- | --- |
| Abujarad et al [44] | Patient centered virtual multimedia interactive IC tool: virtual coach, video clips, animations, audio, presentations, quiz, retrievable electronic record of IC | Hypothetical | Conceptual design, focus groups, usability evaluation (including SUS and computer efficacy scale) |
| Anderson et al [16] | N/A | N/A | Focus groups, survey (including modified versions of the RTS, Research Attitude Questionnaire and SDHC scale) |
| Balestra et al [29] | eIC without social annotations vs eIC with social annotations | Hypothetical | Survey based on Westin's privacy index, genomics tutorial comprising a quiz |
| Balestra et al [30] | eIC with positive, negative or mixed-valence social annotations | Hypothetical | Survey based on Westin's privacy index, genomics tutorial comprising a quiz |
| Bobb et al [27] | Telemedicine-enabled vs face-to-face consent | Real | Survey including the modified QuIC instrument |
| Budin-Ljøsne et al [35] | N/A | N/A | Two-day workshop |
| Bunnell et al [57] | N/A | N/A | Survey |
| Chen et al [41] | REDCap-based eIC: possibility for changing consent elections and withdrawing consent | Real | REDCap |
| Chhin et al [53] | eIC | Real | Survey |
| Doerr et al [46] | eIC: 5-question summative evaluation | Real | Survey and question: “In what ways would you improve or change mPower?" |
| Furberg et al [47] | eIC: multiple-choice quiz questions, interactive narratives | Hypothetical | Interview, survey (including MacCAT-CR) |
| Ham et al [31] | Paper-based IC vs eIC: pictures, illustrations, animations, video clips | Real | Survey using a visual analogue scale |
| Harle et al [40] | eIC application: 19 hyperlinks and 4 pages of information, bullet points | Hypothetical | Interview |
| Harle et al [26] | Interactive trust-enhanced eIC: hyperlinks, trust-enhanced messages vs interactive-only eIC: hyperlinks vs standard eIC | Hypothetical | Survey (including SDHC, subscale of the QuIC instrument, Decision-Making Control Instrument and RTS) |
| Harmell et al [32] | Paper-based IC vs web-media enhanced IC: video clips, static images/graphics, bullet pointed text | Hypothetical | Survey (including UBACC and MacCAT-CR) |
| Haussen et al [60] | REDCap-based eIC | Real | Survey |
| Iwaya et al [43] | eIC: icons, hyperlinks and quiz | Hypothetical | Interview |
| Jayasinghe et al [45] | eIC: 39 separate screen views, large font size, audio narration of the text in a human voice, definitions, illustrations, multiple choice self-test, colored text vs paper-based IC: nine pages long | Hypothetical | Focus group, pilot study (including UBACC) |
| Kane et al [59] | Case scenario with a multimedia consent module | Hypothetical | Survey |
| Kim et al [58] | Tiered eIC: help texts, definitions | Hypothetical | Survey |
| Madathil et al [51] | Paper-based IC vs Topaz-based eIC vs touchscreen-based eIC vs iPad-based eIC | Hypothetical | Survey (including IBM Computer Systems Usability Questionnaire), think-aloud session |
| Mahnke et al [50] | eIC prototype | Hypothetical | Simulation of consents, readability analysis, survey (including SUS), (hybrid) focus group, usability evaluation |
| McGowan et al [56] | eIC: progress bar | Real | Survey |
| Moran-Sanchez et al [52] | eIC: slide show, bulleted much-simplified format, 1 key point per slide, staff member read aloud each presentation slide, large text size | Hypothetical | Survey (including Global Assessment Functional Scale and adapted version of MacCAT-CR), interview |
| Perrault et al [55] | eIC with 71 words, four sentences vs eIC with 300 words | Hypothetical | Survey |
| Ramos et al [49] | eIC: 16 screens, a five-icon home page, highlighted icons with simplified text, colored background and paper IC | Hypothetical | Icon selection (interview), post-test design (interview, survey) |
| Rothwell et al [33] | eIC: video presentation, photographs, graphics and animations vs simplified paper-based consent | Real | Survey, interview |
| Rowan et al [42] | eIC | Hypothetical | Observation, focus group |
| Rowbotham et al [34] | Paper IC vs eIC: video, audio, interactive quiz | Hypothetical | Survey |
| Schneiderheinze et al [54] | eIC: text and audio mode, animated pictograms, hyperlinks | Hypothetical | Survey (including SUS) |
| Simon et al [28] | Standard interactivity paper IC vs enhanced interactivity paper IC: interactive questions vs standard interactivity eIC: graphics, audio vs enhanced interactivity eIC: graphics, audio, interactive questions | Real | Survey |
| Simon et al [39] | Paper IC and eIC: web-based slideshow | Hypothetical | Focus group, survey |
| Simon et al [61] | N/A | N/A | Survey |
| Spencer et al [38] | eIC prototype: hyperlinks | Hypothetical | Interview, focus group |
| Sundby et al [62] | Explanatory video films with subtitles and voice-over | N/A | Survey |
| Tait et al [48] | Child version eIC: 3D modelled animated characters, clicking on icons to receive information about the trial and adult version eIC: live-action elements in an animated 3D office, clicking on icons to receive information about the trial | Hypothetical | Interview, survey |
| Teare et al [37] | eIC mock-up: modifiable information options | Hypothetical | Focus group, interviews |
| Vanaken et al [15] | N/A | N/A | Survey, focus group, interview, meeting |
| Warriner et al [25] | eIC: audio, animated video, avatars, comprehension multiple choice questions vs paper IC | Hypothetical | Survey (including Health Information Technology Usability Evaluation Scale and QuIC instrument) |
| Wood et al [36] | Web pages designed to achieve IC | Hypothetical | Interview |

Abbreviations

eIC: Electronic informed consent

IC: Informed consent

MacCAT-CR: MacArthur Competence Assessment Tool for Clinical Research

N/A: Not applicable

QuIC: Quality of Informed Consent

RTS: Researcher Trust scale

SDHC: Satisfaction with Decisions in Health Care

SUS: System Usability Scale

UBACC: University of California, San Diego Brief Assessment of Capacity to Consent
